# Pro-fibrotic and apoptotic activities of circARAP1 in myocardial ischemia–reperfusion injury

**DOI:** 10.1186/s40001-023-01001-0

**Published:** 2023-02-20

**Authors:** Xi Li, Lei Guo, Jingjing Wang, Xing Yang

**Affiliations:** 1 Department of Cardiology, General Hospital of Ningxia Medical University, No. 804, Shengli South Street, Xingqing District, Yinchuan, 750003 Ningxia Hui Autonomous Region China; 2 Department of Cardiology, Yan’an University Xianyang Hospital, Xianyang, 716099 Shaanxi China

**Keywords:** Myocardial ischemia–reperfusion injury, circARPA1, miR-379-5p, KLF9, Wnt pathway

## Abstract

Reperfusion modality can cause damage to cardiomyocytes, known as myocardial ischemia–reperfusion injury (MI/RI). Circular RNAs (circRNAs) are fundamental regulators associated with many cardiac diseases, including MI/RI. However, their functional impact on cardiomyocyte fibrosis and apoptosis remains elusive. Therefore, this study aimed to explore possible molecular mechanisms of circARPA1 in animal models and in hypoxia/reoxygenation (H/R)-treated cardiomyocytes. GEO dataset analysis showed that has_circ_0023461 (circARPA1) was differentially expressed in myocardial infarction samples. Real-time quantitative PCR further supported that circARPA1 was expressed at high levels in animal models and in H/R-triggered cardiomyocytes. Then, loss-of-function assays were performed to show that circARAP1 suppression effectively ameliorated cardiomyocyte fibrosis and apoptosis in MI/RI mice. Mechanistic experiments showed that miR-379-5p, KLF9 and Wnt signaling pathways were associated with circARPA1. circARPA1 can sponge miR-379-5p to regulate KLF9 expression, thereby activating the wnt/β-catenin pathway. Finally, gain-of-function assays revealed that circARAP1 aggravated MI/RI in mice and H/R-induced cardiomyocyte injury by regulating the miR-379-5p/KLF9 axis to activate Wnt/β-catenin signaling.

## Introduction

Myocardial infarction (MI) causes high disability and mortality in patients with cardiovascular disease worldwide [1]. Timely and complete reperfusion (primary percutaneous coronary intervention and clinical thrombolysis) can decrease mortality from MI [2, 3], reperfusion itself may lead to hypersensitivity irreversible myocardial injury and cardiac dysfunction, namely myocardial ischemia–reperfusion injury (MI/RI) [4]. MI/RI can lead to a series of adverse effects and damages, such as reinfarction, malignant arrhythmia and heart failure [5]. Currently, the main treatments for MI/RI include ischemic postconditioning, remote ischemic preconditioning, and drug therapy [6]. However, microvascular dysfunction occurs after treatment [7, 8]. Protecting the heart from the deleterious effects of MI/RI remains a therapeutic challenge.

Circular RNAs (circRNAs) are endogenous transcripts that are expressed wildly [9] and implicated in multiple cardiovascular diseases [10, 11]. For example, circFoxo3 inhibits autophagy in MI, thereby inhibiting MI/RI [12]. CircHIPK3 offers autophagic and apoptotic properties in MI/RI [13] and circSAMD4A can promote hypoxia/reoxygenation (H/R)-induced apoptosis and inflammatory responses [14]. GEO dataset (GSE160717) from the circMine website screened out the differentially expressed circRNAs during MI/RI and identified has_circ_0023461 (circARPA1) as the circRNA of interest because of the highest differential expression. However, to date, whether circARPA1 is involved in the development of MI/RI has not been confirmed. Furthermore, the 3′-UTR microRNA (miR)‐379-5p contained a putative binding site for circARPA1 and 3-UTR region of KLF9 contained a putative binding site for miR-379-5p. It has been described that KLF9 aggravates ischemic injury in cardiomyocytes by increasing oxidative stress [15]. Given that, this study investigated the underlying molecular mechanisms of circARPA1 in MI/RI and assessed whether circARPA1 could serve as a therapeutic target for MI/RI.

## Materials and methods

### Cell culture

DMEM (Thermo, USA) consisting of 10% FBS (Gibco, USA) and 1% penicillin/streptomycin was in application to culturing mouse cardiomyocytes HL-1 (ATCC, USA). The in vitro MI/RI model was established on cardiomyocytes cultured for 6 h under conditions of 1% O_2_, 5% CO_2_ and 94% N_2_, and then for 4 h under conditions of 95% air and 5% CO_2_.

### Cell transfection

siRNA or pcDNA 3.1 overexpression vectors targeting circAPAR1 and KLF9, miR-379-5p mimic/inhibitor and their negative controls were designed and provided by GenePharma (Shanghai, China). Following H/R, the above plasmids or oligonucleotides were transfected into HL-1 cells using Lipofectamine 2000 (Invitrogen, USA) and the transfection efficiency was assessed after 48 h. To inhibit the Wnt pathway, cells were pretreated with 10 μM ICG-001 for 0.5 h before transfection.

### RNase R test

Trizol method-based extraction of total RNA (4 μg) was performed, followed by RNase R digestion (4 U/μg) for 30 min. The products were analyzed using RT-qPCR.

### Actinomycin D test

HL-1 cells were incubated with 2 mg/ml actinomycin D (Sigma-Aldrich, MO, USA), followed by measurements of circRNA or mRNA at different time points (0, 4, 8, 12, 24).

### Flow cytometry

Apoptosis detection was performed on HL-1 cells (1 × 10^6^ cells/ml) based on annexin V-fluorescein isothiocyanate/propidium iodide staining kit (Solarbio, China) and data acquisition was completed on a FACS Calibur flow cytometer (BD Biosciences).

### ROS detection

ROS production was calculated by 2′,7′-dichlorofluorescein diacetate staining (Sigma, USA). Fluorescence intensity was analyzed by flow cytometry.

### LDH release and creatine kinase (CK) detection

After I/R modeling, serum samples were separated from the femoral arterial blood of mice. LDH and CK in mouse serum and HL-1 cell supernatant were analyzed by LDH kit (C0017, Beyotime Biotechnology, Shanghai, China) and CK kit (ML-ELISA-0252, R&D Systems).

### FISH assay

Subcellular localization of circARAP1 and miR-379-5p was determined using fluorescent probes for Cy3-labeled circARAP1 and Fam-labeled miR-379-5p (Servicebio, Wuhan, China) and FISH kits (Roche, Basel, Switzerland). Cells were first fixed with 4% paraformaldehyde, permeabilized with 0.25% Triton X-100, hybridized with specific labeled probes, and stained with DAPI. Images were taken under a fluorescence microscope (BX53, Olympus).

### Nucleocytoplasmic separation

Cytoplasmic and nuclear RNAs were isolated using the PARIS kit (Life Technologies) and conditioned to circARAP1 quantitative analysis.

### circRIP and RIP experiments

circRIP assay was performed with BersinBio^™^ RIP kit (BersinBio, Guangzhou, China) [16] and RIP assay was with EZ-Magna RIP kit (Millipore). HL-1 cells with biotin-NC and biotin-miR-379-5p were lysed in complete RIP lysis buffer and combined with mouse anti-Ago2-conjugated magnetic beads in RIP buffer. Finally, RNA was extracted by the phenol–chloroform method and quantified.

### Dual luciferase reporter assay

circARAP1/KLF9 sequences containing wild-type or mutant miR-379-5p binding sites were synthesized and inserted into pmirGLO luciferase vector (GeneCreate, Wuhan, China). HL-1 cells (5 × 10^3^ cells/well) were placed in a co-transfection system containing miR-379-5p mimic or negative control and vectors based on Lipofectamine 2000 to examine luciferase activity using a dual-luciferase assay system (Promega).

### MI/RI modeling

A total of 75 healthy male C57BL/6 mice (6–8 weeks old, 20–30 g) were purchased from SJA Laboratory Animal Co., Ltd. and adaptively housed for 1 week before MI/RI modeling [17]. Briefly, the thoracic cavity was exposed after intraperitoneal injection of pentobarbital sodium (30 mg/kg), and the left anterior descending artery (LAD) was ligated with silk 6-0. Successful myocardial ischemia, such as myocardial discoloration, decreased pulse, and ST-segment elevation, was observed on the ECG. After 30 min of ischemia, the ligatures were untied to restore blood flow for 120 min. Sham operation excluded LAD ligation. An overexpression vector targeting circARAP1 (oe-circARAP1) and a negative control vector (oe-NC) were injected via tail vein (50 nM) one week before surgery. Wnt signaling inhibitor ICG-001 (5 mg/kg/day, Chembest, China) was injected via tail vein for 5 days after MI/R surgery. After 10 days, the mice were euthanized, 5 mouse hearts in each group were used for TTC staining, and the rest was for routine pathological staining and gene extraction.

### Cardiac function

LVDP, LVSP, and ± dp/dtmax were recorded by RM6240B multi-channel physiological signal acquisition and processing system 7 days after MI/RI surgery.

### TTC staining

TTC staining was used to assess infarct size. Quickly move the heart to a − 80 °C refrigerator for 20 min. Frozen hearts were cut transversely into 2-mm sections for dyeing with 1% TTC (Sigma-Aldrich). After 4% paraformaldehyde treatment, tissue sections were photographed with a digital camera (Nikon, Tokyo, Japan) and analyzed by Image J software.

### HE and Masson staining

Hearts after paraffin-embedding were cut into 4 μm for H&E staining (Beyotime) and Masson staining (Solarbio). After staining, the sections were photographed under a microscope.

### TUNEL staining

TUNEL staining was done based on the instructions of in situ cell death detection kit (Roche) and DAPI nuclei staining [18].

### IHC staining

After deparaffinization and hydration, sections were blocked with 3% hydrogen peroxide, followed by treatments with 10% normal goat serum (Cat. No. 16210072; Gibco; Thermo Fisher Scientific), primary antibodies α-SMA (A2547, MilliporeSigma), Collagen I (14695–1-AP, Proteintech), Collagen II (15943–1-AP, Proteintech), and HRP-conjugated goat anti-rabbit secondary antibody (1:400; Cat. No. A32731; Invitrogen). After DAB treatment, microscopic images were taken.

### RT-qPCR

Total RNA was obtained using the TRIzol RNA isolation system (Life Technologies) and prepared into first-strand cDNA using a reverse transcription system kit (Promega). RT-qPCR was performed in the ABI PRISM 7000 Sequence Detection System (Applied Biosystems, Foster City, CA) in combination with PCR primer sequences (Table 1). Relative gene expression was determined by the 2^−ΔΔCT^ method [19].

**Table 1:** Primer sequences

Genes	Primer sequences (5′–3′)
circARPA1	Forward: 5′- GTGTTTAGTTGGCGGATGGC-3′
	Reverse: 5′- TTGATGACTGGTGTGACGGG-3′
miR-379-5p	Forward: 5′- GCGCGTGGTAGACTATGGAA -3′
	Reverse: 5′- GCAGGGTCCGAGGTATTC -3′
KLF9	Forward: 5′- TCTGGAGAGTCCCGATGAGG-3′
	Reverse: 5′- GAAAGGGCCGTTCACCTGTA-3′
Collagen I	Forward: 5′- TTCTCCTGGCAAAGACGGAC-3′
	Reverse: 5′- AGTGGCACATCTTGAGGTCG-3′
Collagen II	Forward: 5′- AGAGGCTTTGATGGACGCAA-3′
	Reverse: 5′- CCACCAGGACTGCCGTTATT-3′
U6	Forward: 5′-CTCGCTTCGGCAGCACA-3′
	Reverse: 5′-AACGCTTCACGAATTTGCGT-3′
GAPDH	Forward: 5′- CATCAACGGGAAGCCCATC
	Reverse: 5′- CTCGTGGTTCACACCCATC

Note: circARPA1, circular RNA ARPA1; miR-379-5p, microRNA-379-5p; KLF9, Kruppel-like factor 9; GAPDH, glyceraldehydes-3-phosphate dehydrogenase

### Western blot

Cells or heart tissues were lysed in ice-cold RIPA lysis buffer (Solarbio) containing protease inhibitors (Roche) and proteins were separated by 12% SDS-PAGE and transferred to nitrocellulose membranes. Membranes were prepared against α-SMA (A2547, MilliporeSigma), Collagen I (14695–1-AP, Proteintech), Collagen II (15943–1-AP, Proteintech), Wnt1 (ab15251, Abcam), β-catenin (ab32572, Abcam), KLF9 (ab227920, Abcam), cleaved caspase-3 (ab2302, Abcam), Bax (ab32503, Abcam), GAPDH (60004–1-Ig, Proteintech), as well as HPR-conjugated secondary antibody. With Pierce® ECL Western Blot Substrate (Pierce), protein bands were visualized and analyzed by Image J.

### Data analysis

Statistical analysis of data with at least three biological replicates was performed using SPSS 21.0. Measurement data were shown as mean ± standard deviation (SD) and evaluated by unpaired t-test for bilateral data or one-way analysis of variance and Tukey’s post hoc test for multiple data. *P* < 0.05 was considered statistically significant.

## Results

### CircARAP1 is abnormally overexpressed in MI/RI

**Fig. 1:**
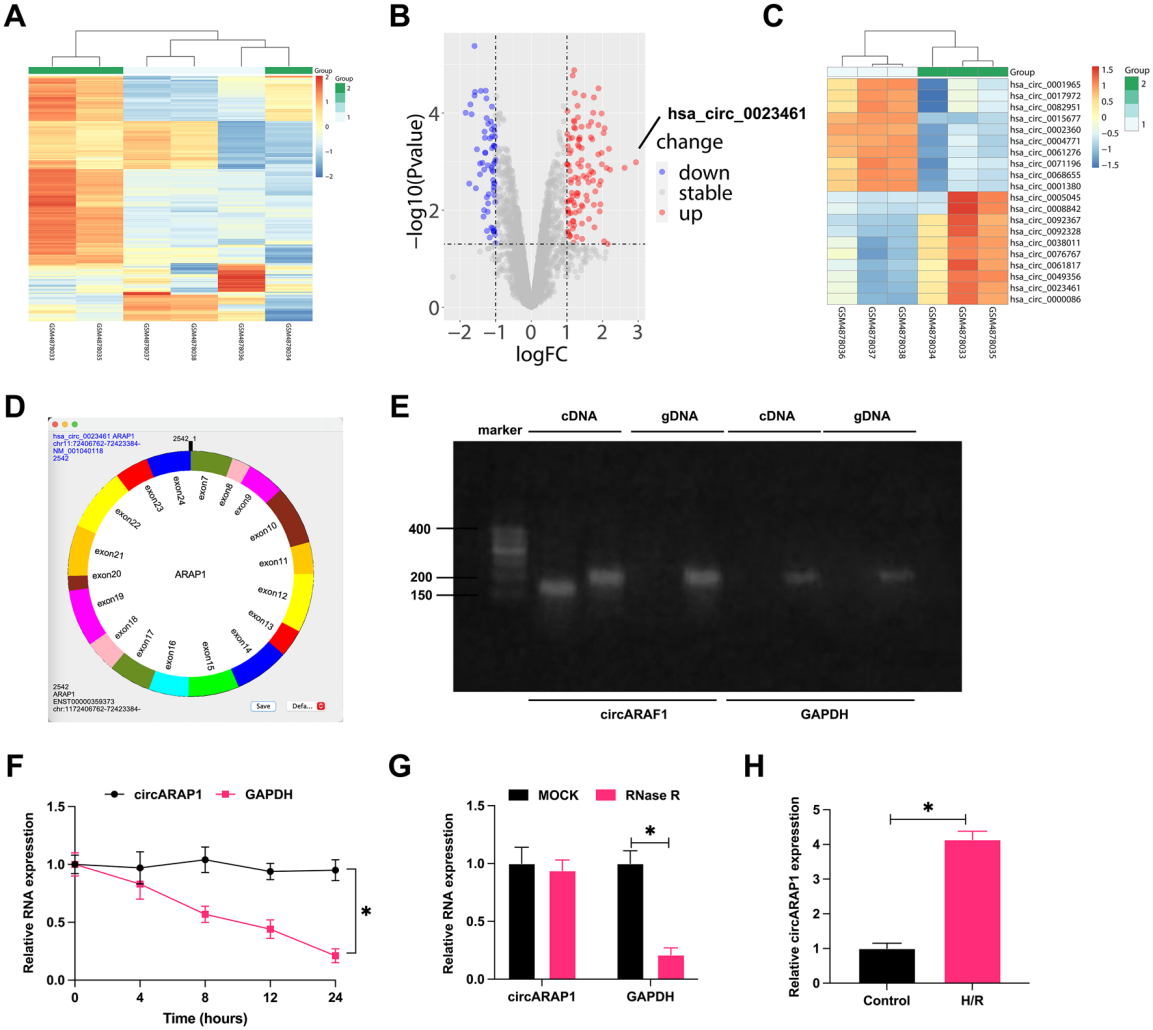
CircARAP1 is abnormally highly expressed in MI/RI. **A**: Heat map showing the top 250 differential circRNAs; **B**: volcano plot of differential circRNAs; **C**: top ten highly expressed and low expressed circRNAs; **D**: gene information of circARAP1; **E**: circular structure of circARAP1; **F** and **G**: ring structure of circARAP1; H: circARAP1 expression in H/R-treated HL-1 cells; data are expressed as mean ± SD (*N* = 3)

To screen out the differential circRNAs during MI/RI, 3 MI samples and 3 normal samples from the GEO dataset (GSE160717) were analyzed through the circMine website. A total of 107 up-regulated and 63 down-regulated circRNAs were found (Supplementary Table 1), of which hsa_circ_0023461 (circARPA1) was identified as the circRNA of interest as having the highest differential expression (log2-fold change = 2.9385) (Fig. 1A–C). Subsequently, gene information of hsacirc0023461 was found on circbase, which is a circular transcript spliced from ARAP1 with a length of 2542 bp, and the gene is located at chr11: 72406762–72423384 (Fig. 1D). Nucleic acid electrophoresis was implemented on the cDNA and gDNA obtained in HL-1 cells and circARAP1 was amplified by different primers in the cDNA, but not the gDNA (Fig. 1E). RNase R could not digest circARAP1, and actinomycin D could not affect the stability of circARAP1 (Fig. 1F, G), further confirming the ring structure of circARAP1. circARAP1 was abnormally highly expressed in H/R-treated HL-1 cells (Fig. 1H).

### Knockdown of circARAP1 improves cardiomyocyte fibrosis and apoptosis

**Fig. 2:**
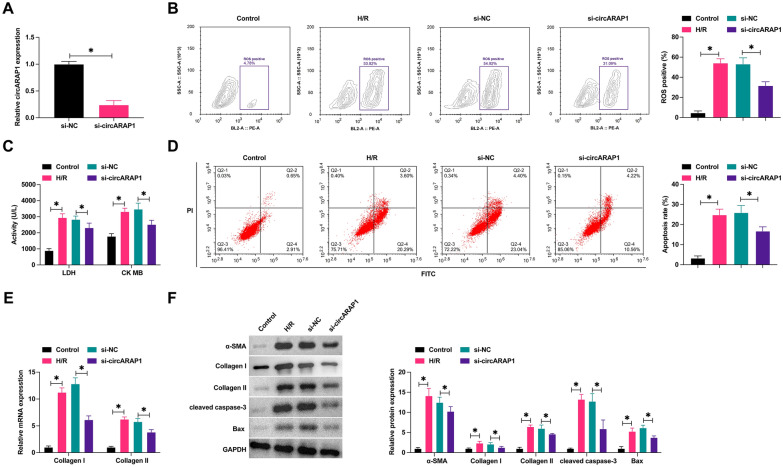
Knockdown of circARAP1 improves cardiomyocyte fibrosis and apoptosis. SiRNA targeting circARAP1 was transfected into H/R-treated HL-1 cells. **A**: circARAP1 expression changes; **B**: ROS production; **C**: LDH and CK levels in HL-1 cells; **D**: apoptosis rate; **E**: Collagen I and Collagen II mRNA in cells; **F**: protein levels of α-SMA, Collagen I, Collagen II, cleaved caspase-3, Bax; data are expressed as mean ± SD (*N* = 3)

To determine the biological function of circARAP1, circARAP1-targeting siRNA was transfected into HL-1 cells, resulting in the decrease of circARAP1 expression in H/R-injured HL-1 cells (Fig. 2A). In H/R-induced ROS production (Fig. 2B), increased levels of LDH and CK (Fig. 2C), and stimulated apoptosis (Fig. 2D). Also, H/R induced an increase in Collagen I and Collagen II mRNA expression (Fig. 2E). Similar results were obtained by Western blot. H/R treatment increased the protein levels of α-SMA, Collagen I, Collagen II, cleaved caspase-3, and Bax (Fig. 2F). The above changes in H/R-treated HL-1 cells were prevented when circARAP1 was knocked down (Fig. 2B–F).

### CircARAP1 targets miR-379-5p

**Fig. 3:**
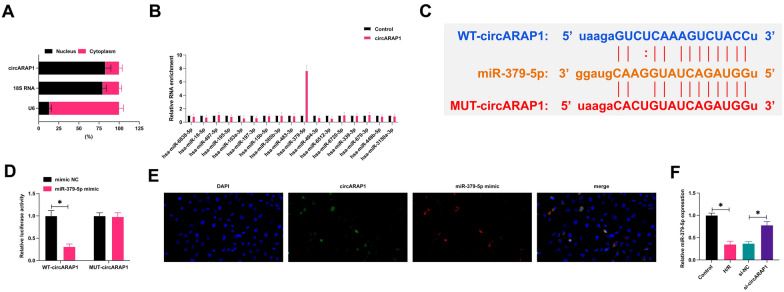
CircARAP1 targets miR-379-5p. **A**: Subcellular localization of circARAP1; **B**: miRNA adsorbed by circARAP1 purified by circRIP; **C**: potential binding site of circARAP1 and miR-379-5p; **D**: targeting relationship between circARAP1 and miR-379-5p; **E**: co-localization of circARAP1 and miR-379-5p in HL-1 cells; **F**: miR-379-5p expression in cells; data are expressed as mean ± SD (*N* = 3)

CircARAP1 was mainly present in the cytoplasm of HL-1 (Fig. 3A). CircARAP1-adsorbed miRNAs were purified by circRIP experiments, among which 17 miRNAs had potential binding sites with circARAP1 on the bioinformatics website, and particularly circARAP1 was highly specifically enriched with miR-379-5p (Fig. 3B). Subsequently, based on the predicted binding sites from https://starbase.sysu.edu.cn (Fig. 3C), WT-circARAP1 and MUT-circARAP1 luciferase reporters were generated, and co-transfection of MUT-circARAP1 and miR-379-5p mimic did not affect luciferase activity, but luciferase activity decreased after co-transfection of WT-circARAP1 and miR-379-5p mimic (Fig. 3D). FISH analysis presented that circARAP1 and miR-379-5p co-localized in the cytoplasm of HL-1 cells (Fig. 3E). As demonstrated in Fig. 3F, H/R treatment inhibited miR-379-5p expression, but knockdown of circARAP1 restored miR-379-5p expression.

### CircARAP1 by modifying miR-379-5p improves cardiomyocyte fibrosis and apoptosis

**Fig. 4:**
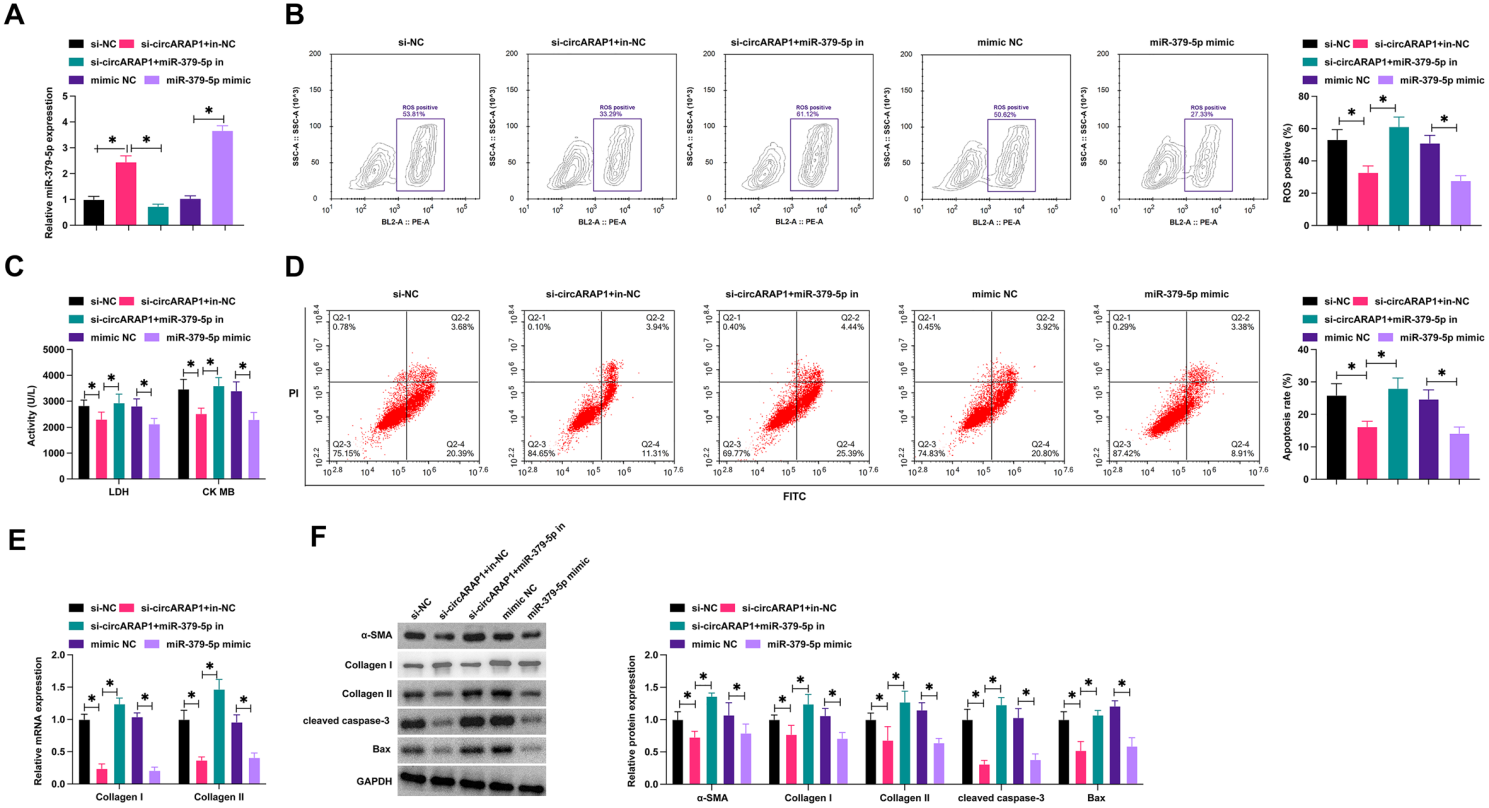
CircARAP1 by modifying miR-379-5p improves cardiomyocyte fibrosis and apoptosis. si-circARAP1, miR-379-5p mimic/inhibitor was transfected into H/R-treated HL-1 cells. **A**: miR-379-5p expression changes; **B**: ROS production; **C**: LDH and CK levels in HL-1 cells; **D**: cell apoptosis rate; **E**: Collagen I and Collagen II mRNA levels; F: protein levels of α-SMA, Collagen I, Collagen II, cleaved caspase-3, Bax; data are expressed as mean ± SD (*N* = 3)

For confirming the biological function of miR-379-5p in MI/RI and whether it is involved in the process of circARAP1 regulating MI/RI, si-circARAP1, miR-379-5p mimic/inhibitor were transfected into H/ R-treated HL-1 cells. si-ARAP1 or miR-379-5p mimic up-regulated miR-379-5p, while the effect of si-circARAP1 was antagonized by miR-379-5p inhibitor (Fig. 4A). In addition, silencing circARAP1 or inducing miR-379-5p exhibited beneficial effects on ameliorating H/R injury, mainly manifested as decreased ROS production, decreased LDH, CK and apoptosis rates, and decreased collagen I/II mRNA level, inhibited α-SMA, collagen I/II, cleaved caspase-3 and Bax protein expression, while the effect of knockdown of circARAP1 was blocked when miR-379-5p was inhibited (Fig. 4B–F).

### KLF9 is a target gene of miR-379-5p

**Fig. 5:**
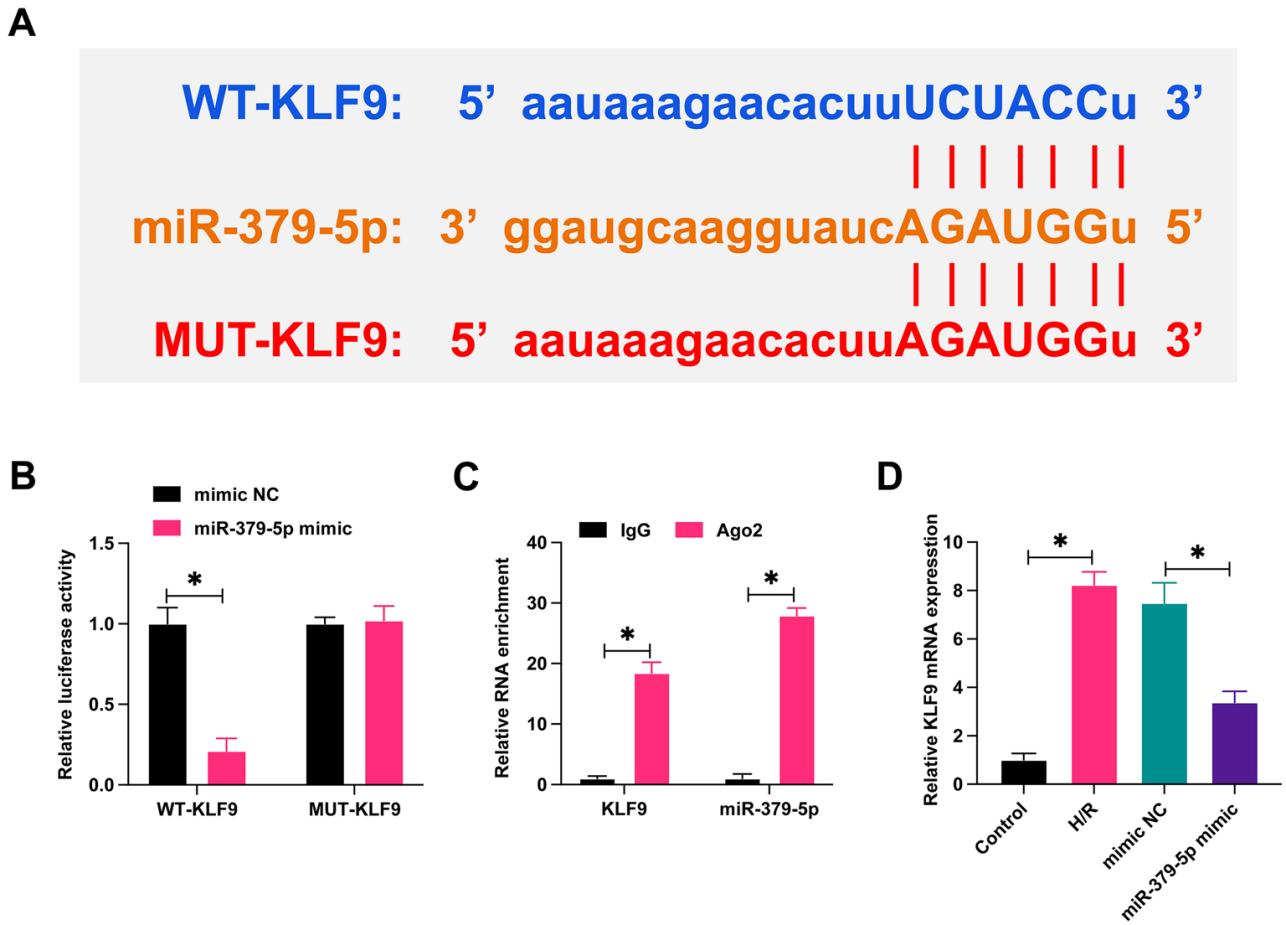
KLF9 is a target gene of miR-379-5p. **A**: Potential binding sites of KLF9 and miR-379-5p; **B**: targeting relationship of KLF9 and miR-379-5p; **C**: binding relationship of KLF9 and miR-379-5p; **D**: expression changes of KLF9 in H/R-treated HL-1 cells; data are expressed as mean ± SD (*N* = 3)

We queried the potential downstream targets of KLF9 and miR-379-5p from the bioinformatics website https://starbase.sysu.edu.cn (Fig. 5A). Subsequently, wild-type and mutant KLF9 luciferase reporter vectors were designed according to potential binding sites and the targeted binding of KLF9 and miR-379-5p was confirmed by dual-luciferase reporter experiments (Fig. 5B). RIP experiments further confirmed that KLF9 and miR-379-5p were significantly enriched (Fig. 5C). By RT-qPCR, it was found that H/R treatment induced an increase in KLF9 expression, whereas overexpression of miR-379-5p prevented this change (Fig. 5D).

### CircARAP1 ameliorates cardiomyocyte fibrosis and apoptosis by regulating the miR-379-5p/KLF9 axis

**Fig. 6:**
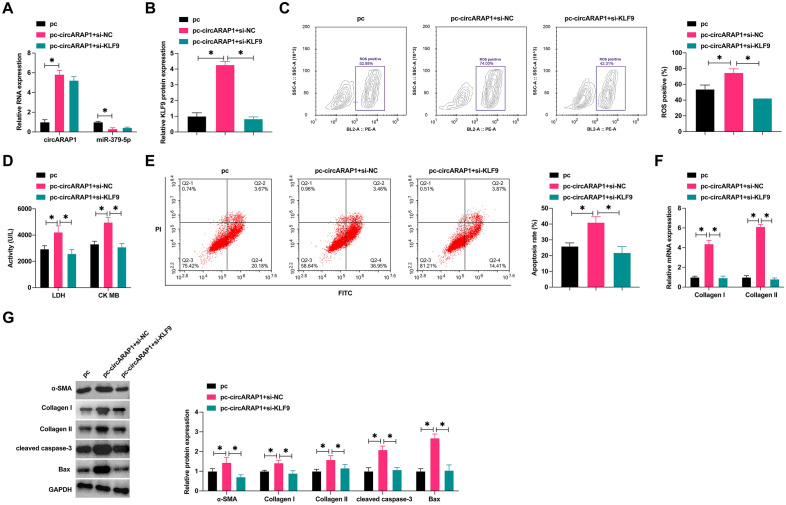
CircARAP1 improves cardiomyocyte fibrosis and apoptosis by regulating miR-379-5p/KLF9 axis. pcDNA 3.1-circARAP1 and si-KLF9 were co-transfected into H/R-treated HL-1 cells. **A**–**B**: circARAP1, miR-379-5p, and KLF9 expression changes; **C**: ROS production; **D**: LDH and CK in HL-1 cells; **E**: apoptosis rate; **F**: Collagen I and Collagen II mRNA levels; **G**: protein levels of α-SMA, Collagen I, Collagen II, cleaved caspase-3 and Bax level; data are expressed as mean ± SD (*N* = 3)

Subsequently, pcDNA 3.1-circARAP1 and si-KLF9 were co-introduced into H/R-treated HL-1 cells. pcDNA 3.1-circARAP1 increased circARAP1 and KLF9 but suppressed miR-379-5p levels, while si-KLF9 decreased KLF9 expression but did not affect circARAP1 and miR-379-5p expression on the basis of pcDNA 3.1-circARAP1 (Fig. 6A, B). circARAP1 induction further aggravated H/R-induced cardiomyocyte fibrosis and apoptosis, and depletion of KLF9 reversed these phenomena (Fig. 6C–G).

### CircARAP1/miR-379-5p/KLF9 axis regulates the Wnt/β-catenin pathway

**Fig. 7:**
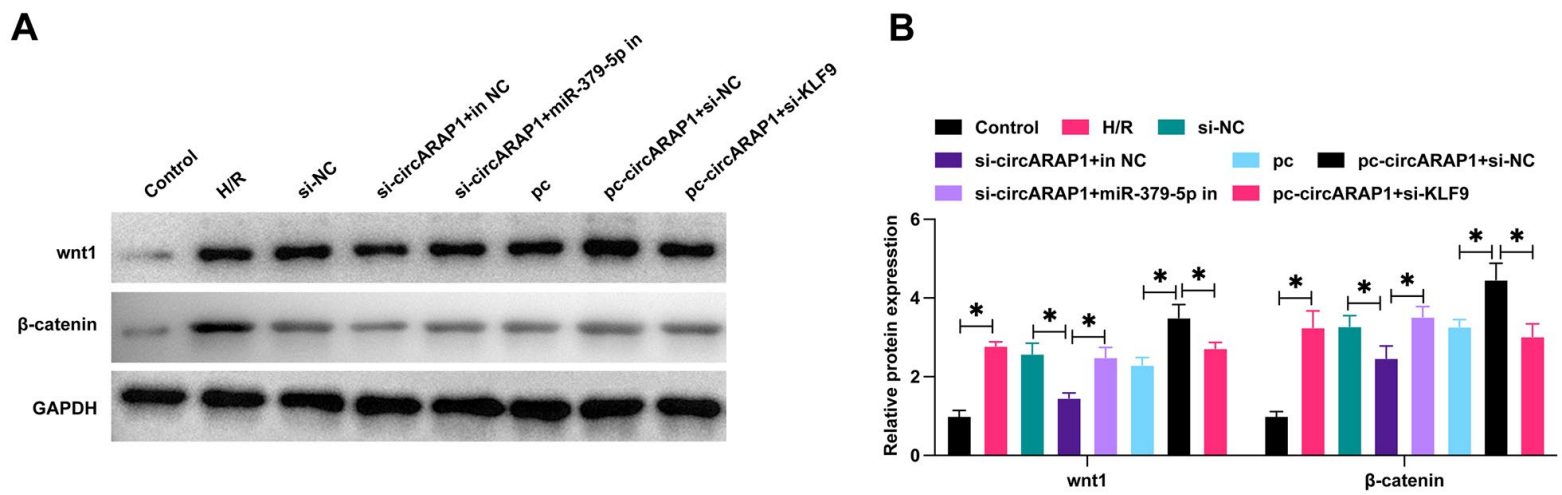
CircARAP1/miR-379-5p/KLF9 axis regulates Wnt/β-catenin pathway. **A**–**B**: Protein expressions of Wnt1 and β-catenin in HL-1 cells; data are expressed as mean ± SD (*N* = 3)

Wnt/β-catenin pathway is abnormally activated in I/RI and exacerbates I/RI [20–22]. Therefore, this study speculates that circARAP1 may activate the Wnt/β-catenin pathway by affecting the miR-379-5p/KLF9 axis. To test this notion, the protein changes in Wnt1 and β-catenin were tested by western blot. As predicted, H/R treatment activated the Wnt/β-catenin pathway, knockdown or overexpression of circARAP1 prevented and promoted the activation of the Wnt/β-catenin pathway, respectively, which was mitigated by silencing miR-379-5p and KLF9, respectively (Fig. 7A, B).

### Wnt/β-catenin pathway is critical for circARAP1 to affect H/R injury

**Fig. 8:**
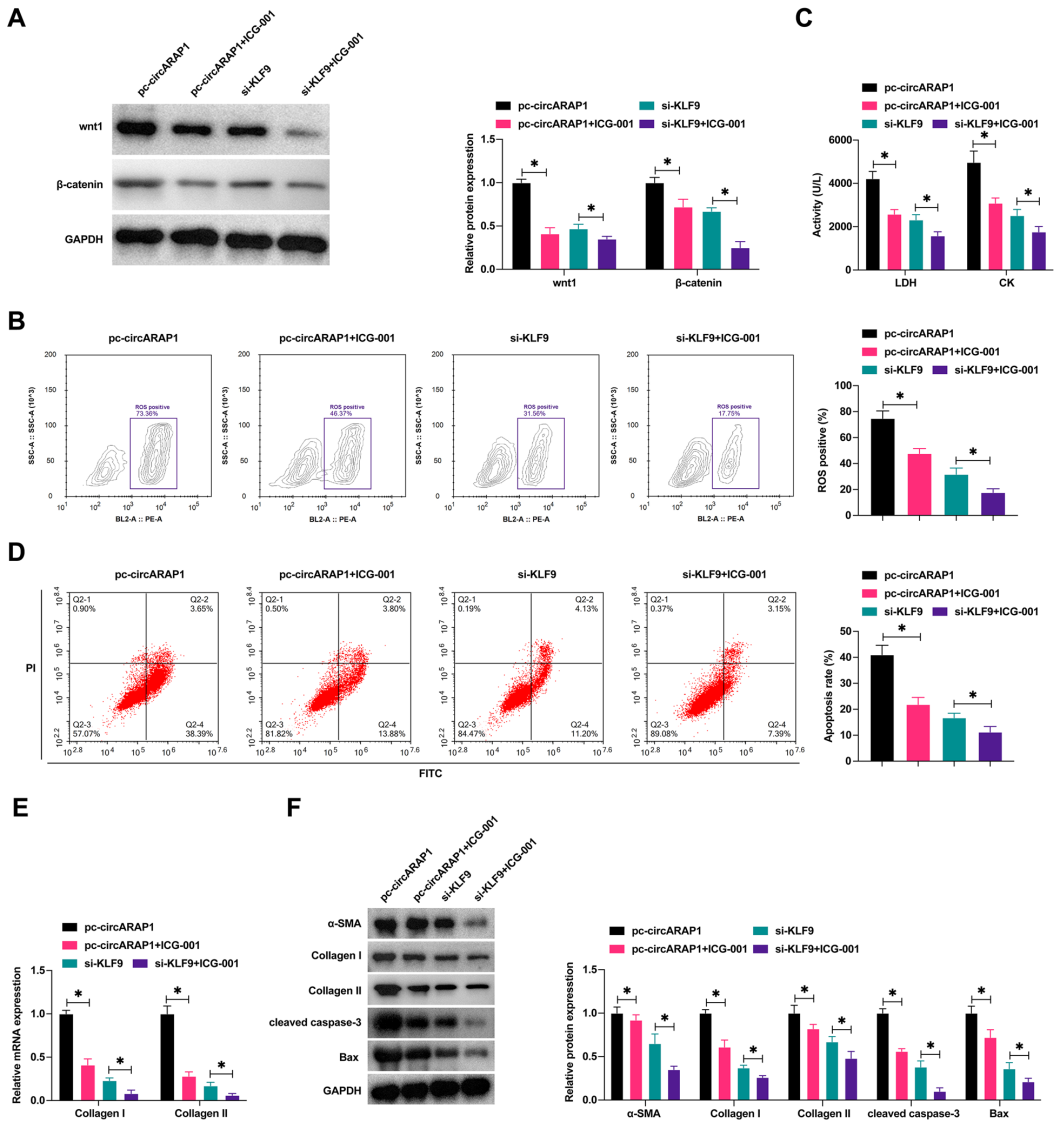
Wnt/β-catenin pathway is critical for circARAP1 to affect H/R injury. ICG-001 blocked the wnt/β-catenin pathway in HL-1 cells overexpressing circARAP1 or knocking down KLF9. **A**: Protein expressions of Wnt1 and β-catenin; **B**: ROS production; **C**: LDH and CK in HL-1 cells; **D**: apoptosis rate; **E**: Collagen I and Collagen II mRNA levels; **F**: protein levels of α-SMA, Collagen I, Collagen II, cleaved caspase-3, Bax; data are expressed as mean ± SD (*N* = 3)

Western blot showed that ICG-001 inhibited wnt/β-catenin signaling in HL-1 cells overexpressing circARAP1 or knocking down KLF9 (Fig. 8A). Functional experiments showed that ICG-001 led to the reduction of ROS production, inhibited LDH, CK, Collagen I/II and decreased apoptosis, while reducing α-SMA, collagen I/II, cleaved caspase-3 and Bax protein expression in HL-1 cells overexpressing circARAP1, and ICG-001 further enhanced the therapeutic effect of KLF9 knockdown on H/R-induced fibrosis and apoptosis (Fig. 8B–F).

### CircARAP1 affects MI/RI in mice by regulating miR-379-5p/KLF9 axis-regulated Wnt/β-catenin pathway

**Fig. 9:**
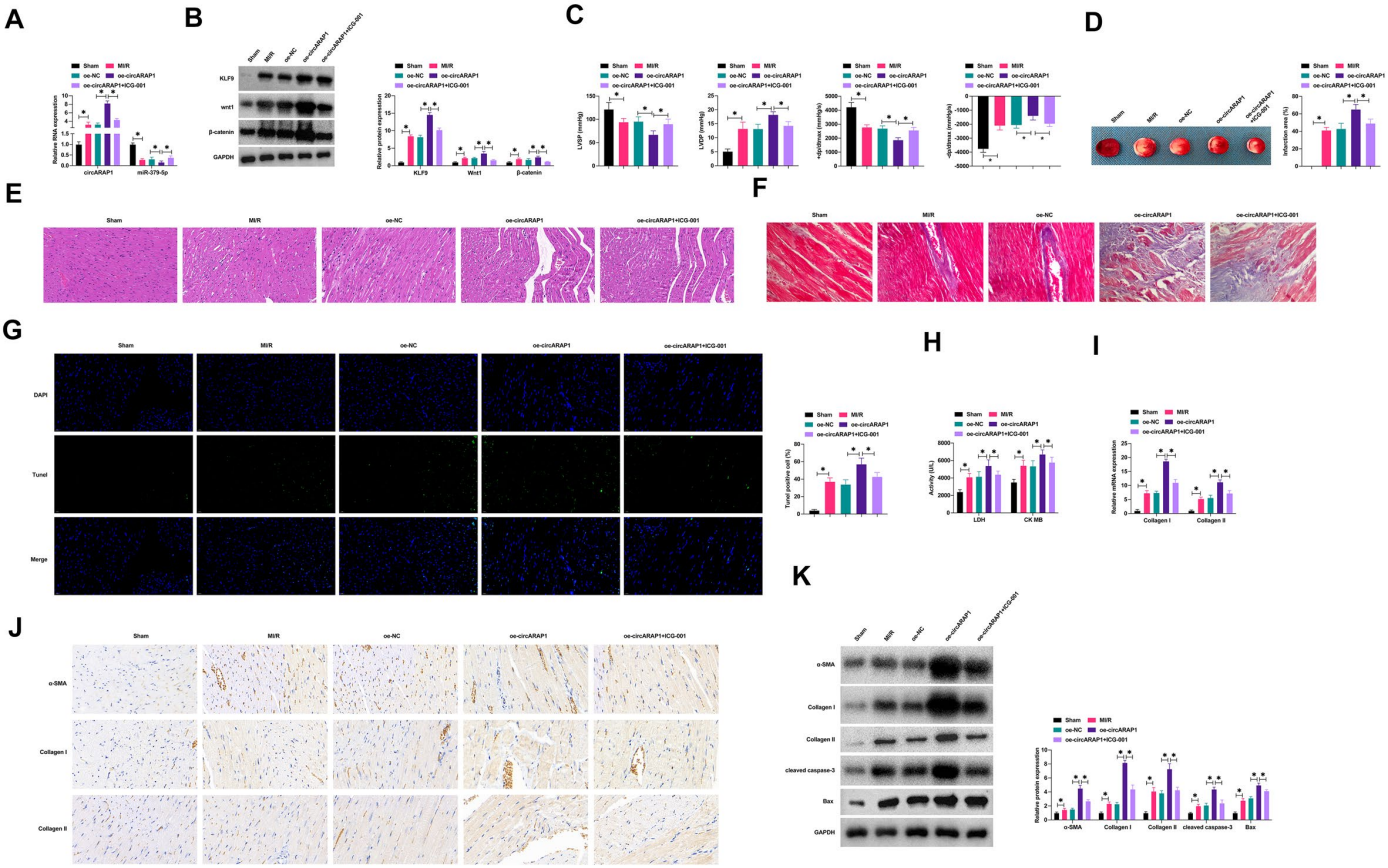
CircARAP1 affects MI/RI in mice by regulating miR-379-5p/KLF9 axis and activating Wnt/β-catenin signaling. Oe-circARAP1 was injected into MI/R-injured mice, and ICG-001 was used to block Wnt/β-catenin signaling. A: circARAP1 and miR-379-5p mRNA levels; **B**: protein expression of KLF9, wnt1 and β-catenin; **C**: mouse cardiac function index; **D**: representative graph of cardiac TTC staining; **E**: representative IMAGES of myocardial tissue HE staining; **F**: representative graph of Masson staining in myocardial tissue; **G**: representative graph of TUNEL staining in myocardial tissue; **H**: serum LDH and CK levels in mice; I: Collagen I/II levels in mouse myocardial tissue; **J**: IHC staining of α-SMA, collagen I/II in myocardial tissue; **K**: protein level of α-SMA, Collagen I, Collagen II, cleaved caspase-3, Bax in myocardial tissue; data are expressed as mean ± SD (D, *n* = 5; **A**–**C**, **E**–**K**, *n* = 10)

Oe-circARAP1 was injected into MI/R-injured mice and ICG-001 was utilized to block Wnt/β-catenin signaling. MI/RI resulted in increased expression of circARAP1, KLF9, wnt1 and β-catenin and decreased expression of miR-379-5p, while oe-circARAP1 further enhanced the effect of H/R on these genes, but ICG-001 attenuated the effect of oe-circARAP1 (Fig. 9A, B). Detection of cardiac function showed that MI/RI mice had higher LVDP, but lower LVSP, ± dp/dtmax, and oe-circARAP1 enhanced this change, but blocking Wnt/β-catenin signaling attenuated this effect of oe-circARAP1 (Fig. 9C). Tissue staining showed that MI/RI mice exhibited larger myocardial infarct size, more severe myocardial tissue damage, more collagen deposition and higher apoptosis rate, oe-circARAP1 aggravated these pathological changes, but blocking the Wnt/β-catenin pathway attenuated the effect of oe-circARAP1 (Fig. 9D–G). Also, LDH, CK, α-SMA, collagen I/II, cleaved caspase-3 and Bax in MI/RI mice were higher, and oe-circARAP1 further up-regulated the levels of these factors, but disruption of Wnt/β-catenin pathway attenuated the effect of oe-circARAP1 (Fig. 9H–K).

## Discussion

CircRNA is a novel regulatory RNA involved in multiple pathological cardiac progression [23]. Here, circARPA1 was identified as the circRNA of interest. However, to date, the role of circARPA1 in MI-induced myocardial injury remains obscure. In the current study, we identified circARAP1 as a circular RNA and found that circARAP1 was abnormally highly expressed in MI/R-injured mice and H/R-induced cardiomyocytes. Furthermore, we identified novel functions and mechanisms of circARPA1 in regulating myocardial fibrosis and apoptosis in MI/RI.

Myocardial apoptosis and fibrosis are one of the main mechanisms in the progression of MI/RI [24, 25]. The earliest and predominant form of infarcted cardiomyocyte death is apoptosis [24]. Cardiac fibrosis is caused by excessive deposition of extracellular matrix and activation of myofibroblasts in damaged areas, leading to scarring and permanent impairment of cardiac function [26]. Therefore, elucidating the underlying mechanisms of myocardial fibrosis and apoptosis and identifying new strategies are crucial for the treatment of MI/RI. Oxidative damage in the myocardium due to the overproduction of ROS exacerbates cardiomyocyte apoptosis and myocardial fibrosis [27, 28]. Here, it was found that H/R treatment promoted ROS production, increased levels of LDH and CK, protected cardiomyocytes by inhibiting apoptosis and fibrosis, as demonstrated by H/R leading to increased cardiomyocyte apoptosis rates and increased expression of fibrotic and apoptotic indicators, while knockdown of circARAP1 reversed these phenomena.

Cytoplasmic circRNAs competitively bind miRNAs, thereby exerting effects on gene regulation [29]. circARAP1 was mainly present in the cytoplasm of HL-1, suggesting that circARAP1 mainly acts as a ceRNA for downstream miRNAs to play a post-transcriptional regulation role on downstream gene expression. miR-379-5p highly specifically enriched with circARAP1 was screened. Most studies on miR-379-5p have focused on cancer research [30–32], and it has been implicated to exhibit neuroprotective effects against ischemic stroke and reduce neuronal autophagy [33]. Furthermore, in the treatment of atherosclerosis, miR-379-5p is involved in growth, migration and invasion of vascular smooth muscle cells [34]. This study performed a functional rescue experiment to reveal that miR-379-5p was beneficial to ameliorate cardiomyocyte fibrosis and apoptosis.

KLF transcription factor family consists of 17 members that can regulate various biological processes, and alterations in their function have been implicated in the pathobiology of many human diseases, including cardiovascular diseases [35]. KLF9, also known as BTE-B1 [36], has recently been demonstrated to aggravate ischemic injury in cardiomyocytes by increasing oxidative stress [15]. Interestingly, this study found that KLF9, a downstream target gene of miR-379-5p, was up-regulated in H/R-induced HL-1 cells. As expected, data analysis supported that circARAP1 ameliorates H/R-induced cardiomyocyte fibrosis and apoptosis by regulating the miR-379-5p/KLF9 axis.

Wnt/β-catenin pathway plays a key role in cell growth, differentiation, migration, polarity and death [37]. Recently, the wnt/β-catenin pathway has been shown to be abnormally activated in I/RI [20–22]. The present study exhibited that circARAP1 activates the Wnt/β-catenin pathway by regulating the miR-379-5p/KLF9 axis to affect cardiomyocyte H/R injury and MI/RI in mice.

Although the effect of circARAP1 on MI/RI was assessed, the clinical significance of circARAP1 in MI/RI should be confirmed in future studies. Also, impacts of circARAP1 on other kinds of cardiomyocytes need to be clarified, such as H9c2 and AC16 cells. Finally, the molecular mechanisms responsible for the production and upregulation of circARAP1 require further in-depth study.

## Conclusion

CircARAP1 aggravates myocardial ischemia-induced fibrosis and apoptosis by regulating the miR-379-5p/KLF9 axis to activate the Wnt/β-catenin pathway, and circARAP1 may be a promising therapeutic mechanism for MI/RI.

## Data Availability

Data are available from the corresponding author on request.
